# Tracking of mesenchymal stem cells labeled with gadolinium diethylenetriamine pentaacetic acid by 7T magnetic resonance imaging in a model of cerebral ischemia

**DOI:** 10.3892/mmr.2014.2805

**Published:** 2014-10-29

**Authors:** KUAN GENG, ZHONG XIAN YANG, DEXIAO HUANG, MEIZI YI, YANLONG JIA, GEN YAN, XIAOFANG CHENG, RENHUA WU

**Affiliations:** 1 The Chinese People’s Liberation Army 59 Hospital, Yunnan, Kaiyuan, Yunnan 661699, P.R. China; 2 Department of Medical Imaging, The Second Affiliated Hospital, Medical College of Shantou University, Shantou, Guangdong 515041, P.R. China; 3 Provincial Key Laboratory of Medical Molecular Imaging, The Second Affiliated Hospital, Medical College of Shantou University, Shantou, Guangdong 515041, P.R. China

**Keywords:** tracking, stem cells, gadolinium diethylenetriamine pentaacetic acid, 7T magnetic resonance imaging

## Abstract

Progress in the development of stem cell and gene therapy requires repeatable and non-invasive techniques to monitor the survival and integration of stem cells *in vivo* with a high temporal and spatial resolution. The purpose of the present study was to examine the feasibility of using the standard contrast agent gadolinium diethylenetriamine pentaacetic acid (Gd-DTPA) to label rat mesenchymal stem cells (MSCs) for stem cell tracking. MSCs, obtained from the bilateral femora of rats, were cultured and propagated. The non-liposomal lipid transfection reagent effectene was then used to induce the intracellular uptake of Gd-DTPA. Electron microscopy was used to detect the distribution of Gd-DTPA particles in the MSCs. The labeling efficiency of the Gd-DTPA particles in the MSCs was determined using spectrophotometry, and MTT and trypan blue exclusion assays were used to evaluate the viability and proliferation of the labeled MSCs. T1-weighted magnetic resonance imaging (MRI) was used to observe the labeled cells *in vitro* and in the rat brain. Gd-DTPA particles were detected inside the MSCs using transmission electron microscopy and a high labeling efficiency was observed. No difference was observed in cell viability or proliferation between the labeled and unlabeled MSCs (P>0.05). In the *in vitro* T1-weighted MRI and in the rat brain, a high signal intensity was observed in the labeled MSCs. The T1-weighted imaging of the labeled cells revealed a significantly higher signal intensity compared with that of the unlabeled cells (P<0.05) and the T1 values were significantly lower. The function of the labeled MSCs demonstrated no change following Gd-DTPA labeling, with no evident adverse effect on cell viability or proliferation. Therefore, a change in MR signal intensity was detected *in vitro* and *in vivo*, suggesting Gd-DTPA can be used to label MSCs for MRI tracking.

## Introduction

Various types of stem cell have been used in the treatment of diseases of the nervous system, including injury (1), stroke (2) and neurodegenerative diseases (3). Bone marrow stem cell therapy has offered promise in the treatment of diseases and injuries of the brain and spinal cord and previous studies have demonstrated functional recovery following a stroke and in Parkinson’s disease using stem cell transplantation (4,5). However, non-invasive methods are required for monitoring transplanted cells following the successful transplantation of cells in clinical therapy. Conventional methods require histological analysis *in vitro*, which cannot be used for the continuous investigation of transplanted cells *in vivo* (6). However, magnetic resonance (MR) scanners can be used for detecting the migration of implanted stem cells. In order to use MR imaging (MRI) to trace stem cells in the brain, incorporation of MRI contrast agents (CAs) into the cells of interest is required. Two main classes of CA are used for this purpose: Paramagnetic substances, which include T1-shortening CAs, including gadolinium (Gd) chelates (7,8), and superparamagnetic particles (T2-shortening CAs) (9–12).

Due to the advantage of having a high sensitivity for cell tracking, T2 CAs have been widely used for the labeling of numerous types of cell (13–18). However, there are several disadvantages in using T2 CAs for cell tracking, associated with the interpretation of images. Firstly, T2 CAs create signal loss, which may be mistaken for physiological conditions, including hemorrhage, blood flow or pockets of air (19–21) or areas containing high levels of endogenous iron, including the liver, spleen or tumors, including melanoma.

Compared with T2 CAs, Gd-based T1 CAs may be more suitable for cell labeling, due to their higher signal (22). Gd-DTPA has been used to successfully label various types of stem cell, including embryonic and neuronal stem cells (23). Compared with iron oxides, the major drawback of T1 CAs, with respect to cell labeling, is their lower sensitivity. Novel large macromolecular Gd-based CAs, gadolinium rhodamine dextran (24), nanoparticles of gadolinium oxide (25) and gadofullerenes (26) have been identified as T1 CAs, which possess higher relaxivities and improved efficacy in labeling stem cells compared with those of small molecular T1 agents.

In the present study, a basic Gd-DTPA-based cell labeling technique was investigated using an effective transfection reagent with low toxicity to label mesenchymal stem cells prior to imaging. Due to the paramagnetism of the labeling agents, the stem cells were detected using MRI. In addition, the effect of labeling on cellular viability, proliferation and differentiation was determined.

## Materials and methods

### Isolation, cultivation and identification of MSCs

MSCs were isolated and expanded from the bilateral femora of adolescent male Sprague-Dawley (SD) rats weighing between 150 and 200 g, as previously described (27). The rats were supplied by the Shantou University Medical College Laboratory Animal Center, and their age was 7–8 weeks. Briefly, the bilateral femora and tibia were harvested and the marrow was flushed out using a syringe filled with Dulbecco’s modified Eagle’s medium (DMEM)/F12 (Gibco, NY, USA) containing 10% fetal bovine serum. The bone marrow was plated into 25-cm^2^ culture flasks and cultured in an atmosphere of 5% CO_2_ at 37°C for 48 h. The supernatant containing non-adherent cells was then removed and fresh medium was added. When the cells reached ~80–90% confluence, they were passaged two to three times by repeated trypsinization (0.25% trypsin/0.02% EDTA) (Beyotime Biotechnology Institute, Haimen, China) for 2–3 min and subsequent replating. The MSCs were identified and characterized by the absence of staining for CD45 (type: PE-CD45), a surface marker of hematopoietic stem, and positive staining for CD29 and CD45 (BD Biosciences, Franklin Lakes, NJ, USA). All experimental and animal handling procedures were approved by the Animal Care and Use Committee of Shantou University (Shantou, China).

### Cell labeling

Gd-DTPA (Magnevist^®^; Bayer HealthCare Pharmaceuticals, Montville, NJ, USA) is the standard clinically used MR CA, which has a molecular weight of 938 Da. The effectene transfection reagent (Qiagen, Hercules, CA, USA), which is a non-liposomal lipid transfection reagent, was used to transfect Gd-DTPA into the MSCs. As a result of their negative charge, when mixed with effectene, the Gd-DTPA particles were encapsulated with the cationic lipids, which were then efficiently transferred into cells. For cell labeling, single-cell suspensions of 1×10^6^ MSCs were prepared. Gd-DTPA (30 μl; 0.5 mol/l) and effectene (25 μl) were added to the MSCs in a 25-cm^2^ tissue culture flask containing 5 ml DMEM/F12 medium according to the manufacturer’s instructions and were incubated for 30 min (28). During labeling, the MSCs (1×10^6^ cells) were cultured in tissue culture flasks. Subsequently, the effectene/Gd-DTPA mixture (55 μl) was added directly to the DMEM/F12 culture medium for 24 h incubation under standard cell culture conditions (37°C, 5% CO_2_). As a negative control, either 30 μl 0.5 M Gd-DTPA or 25 μl effectene were added to the culture medium of 1×10^6^ cells instead of the 55 μl effectene/Gd-DTPA mixture. Cells were incubated under identical conditions for 4 h in a standard cell culture incubator. A total of 1×10^6^ unlabeled cells were used as a blank control, which were incubated in pure culture medium without the addition of any labeling agents.

### Cellular viability and proliferation

Prior to labeling (0 h) and at 24, 48 and 72 h following the initial labeling procedures, the cell viability was determined using trypan blue exclusion assays. Cellular proliferation was determined using an MTT assay, as previously described (29). In brief, 1×10^4^ cells/well were cultured in flat-bottomed 96-well plates. Subsequently, 50% of the MSCs were labeled and the remaining cells remained unlabeled and served as a control. Following labeling, the cells were washed twice using phosphate-buffered saline. Following this, 200 μl complete culture medium was added to each well and the plate was incubated in a CO_2_ incubator at 37°C and 5% CO_2_. Following 12, 24 and 48 h of incubation, 10 samples of the labeled cells and 10 samples of the unlabeled cells were selected for measurement at each time-point. For measurement, 20 μl MTT (Beyotime Biotechnology Institute, Haimen, China) was added at a final concentration of 5 mg/ml in medium and the cells were incubated for 4 h at 37°C and 5% CO_2_. Following incubation, 150 μl dimethylsulfoxide (DMSO; Sigma, St. Louis, MO, USA) was added and the absorbance of the blue formazan product, formed by the enzymatic reduction of MTT by the living cells, was measured at a wavelength of 570 nm (Bio-Tek ELX800, Vermont, USA), with 750 nm as a reference.

### Cell labeling efficacy and electron microscopy

Labeling efficiency was measured using spectrophotometry, in which the quantity of Gd-DTPA particles in the cells was determined using a spectrometer (Zeeman Z-8200; Hitachi, Tokyo, Japan), as previously described (30). Electron microscopy (CM-10; Philips Scientifics, Eindhoven, The Netherlands) was performed at 60–80 kV. The cells were evaluated for any structural changes as a result of the labeling procedure and for the presence and localization of intracellular CA particles.

### In vitro MRI

The labeled and unlabeled MSCs were trypsinized, centrifuged and resuspended using 20 μl culture medium in 1.5-ml eppendorf tubes (800r/m, 3 min). The total number of cells in each tube was 5×10^5^. MRI of these tubes was performed at 7.0 T (Agilent 7T MRI; Agilent Technologies, Inc., Santa Clara, CA, USA). The MR pulse sequences included T1-weighted (repetition time TR=400 ms; Echo time TE=9 ms) two-dimensional fast spin echo sequences with a 128×128 matrix, four acquisitions, a slice thickness of 1 mm and a 30×30-mm field of view as well as T2-weighted sequences (TR=2,600 ms; TR=100 ms). In addition, for measurements of T1, a T1 map was obtained with a mixed inversion-recovery spin echo sequence, which was initiated with an inversion-recovery experiment followed by a spin echo experiment (TR/TE=10,000 ms/10 ms) to obtain the T1 map. This sequence was performed with a field of view of 30×30 mm, a matrix of 256×128 pixels and a section thickness of 1.5 mm. The signal intensities of the cell pellets were observed and T1 maps were calculated using image software without further modification, as described previously (31). Each *in vitro* MSC MRI procedure was repeated eight times.

### Animal brain ischemia/reperfusion model

Adult male SD rats weighing between 250 and 300 g were used to prepare a conventional ischemia model. As described previously (32), ligations of the common carotid artery and external carotid arteries were performed and a suture was placed through the carotid artery into the internal carotid artery.

### Gd-MSC transplantation

Following successful construction of the ischemic model, chloral hydrate (Yulonghaizao Company, Qingdao, China) was used to anesthetize the SD rats (3 ml/kg; intraperitoneally). The SD rats were then administered with an injection of 1×10^6^ (10 μl each) Gd-MSCs stereotactically. The injection site was in the cortical area, adjacent to the right middle cerebral artery, at a depth of ~3.5–5.0 mm. The coordinates were 1–l.5 mm posterior to the bregma and 2–2.5 mm lateral to the midline. None of the rats received immunosuppressants.

### Gd-MSC observation using MRI

In order to examine the migration of Gd-MSCs *in vivo*, 7-Tesla MRI was used to image the cells 1,3, 5 and 7 days after the Gd-MSC injection, using T1-weighted fast spin echo sequences (light/dark cycle 10/14 h, 22–26°C, diet 30–40 g/d, water 60–70 ml/d). Chloral hydrate 1.5ml/kg was used to anesthetize mice intraperitoneally. The scanning parameters were TR/TE 400/9 ms and an average of four. Each image was confirmed by two professionals.

## Results

### MSC culture and identification

**Figure 1:**
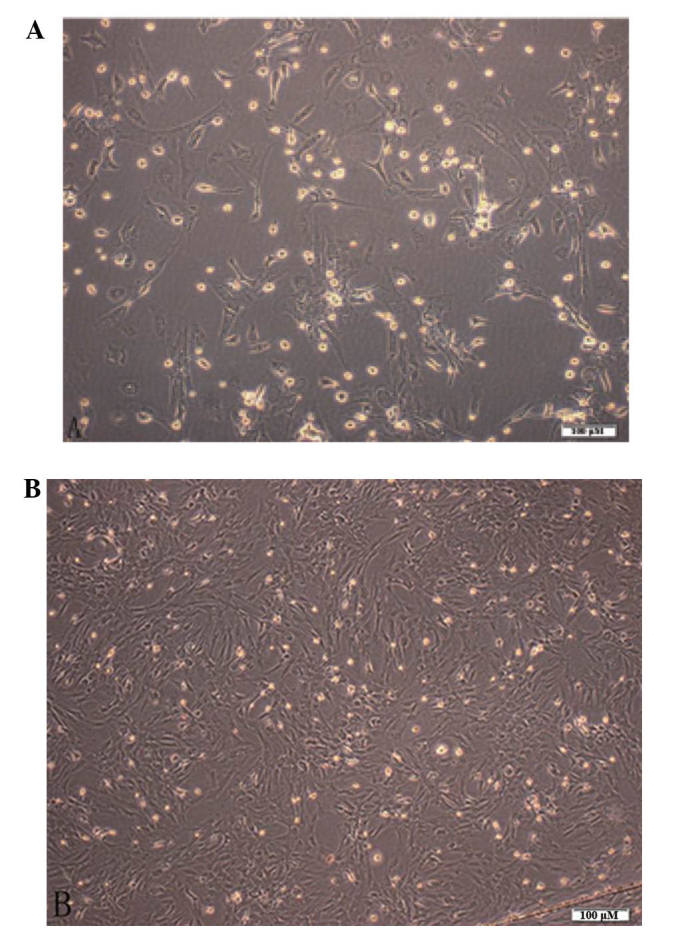
Morphology of the BMSCs (magnification, ×200). (A) Primary BMSCs exhibited a colony-like growth pattern. (B) Passaged BMSCs exhibited a fusiform-like growth pattern. BMSC, bone marrow stromal cell.

**Figure 2:**
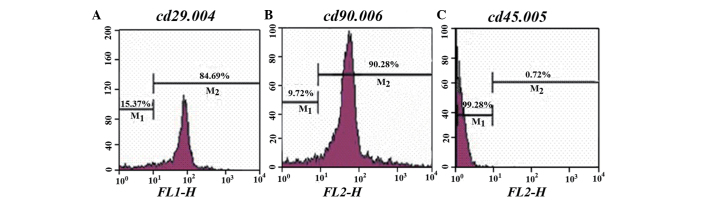
BMSCs were analyzed using fluorescence-activated cell sorting and differentiation assays. Positive rates of expression of the third generation BMSCs of (A) CD29, (B) CD90 and (C) CD45 were 84.69, 90.28 and 0.72, respectively. BMSC, bone marrow stromal cell.

The primary bone marrow stromal cells (BMSCs) were seeded onto petri dishes following spherical suspension in culture medium for 10 h for adherence. After 48 h of adherence, the cells had a stretched appearance with short spindles, triangular centered nuclei and marked refraction, rapidly demonstrating colony of amplification (Fig. 1). The expression of cell surface antigens was detected using flow cytometry. With proceeding incubation time, gradual necrosis was observed in the suspended hematopoietic cells; however, replacing the medium led to the growth of evenly distributed cells with a fusiform fibrotic shape. The expression of cell surface antigens was detected using flow cytometry. The flow cytometeric analysis of the BMSC surface antigens revealed CD29, CD45 and CD90 at 84.69, 0.72 and 90.28%, respectively (Fig. 2).

### Cell labeling

**Figure 3:**
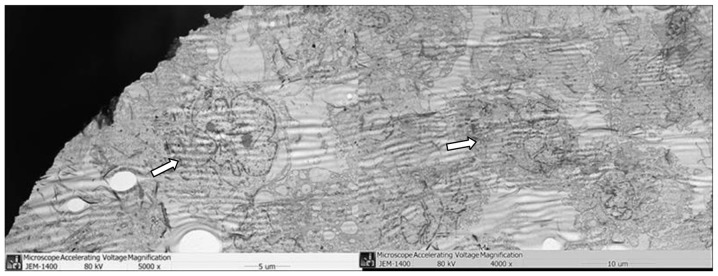
Transmission electron microscopy (magnification, ×50,000) revealed labeled bone marrow stromal cells. Gadolinium diethylenetriamine pentaacetic acid particles were observed within the cytoplasm (arrow).

Gd-DTPA is a small soluble molecule. Following mixing with effectene, numerous Gd-DTPA particles with a negative charge were observed, which were initially condensed together and were subsequently coated with cationic lipids and transferred into cells. The internalized Gd-DTPA particles were mainly aggregated together. Following labeling, the Gd-DTPA particles were clearly visible inside the cytoplasm and accentuated around the cellular apparatus. The high-density particles measured ~50 μm in diameter under the electron microscope (Fig. 3). Using atomic emission spectrophotometry, the effectene-mediated Gd-DTPA labeling efficiency was determined to be 86%.

### Effect of labeling on the biological behavior of the cells

**Figure 4:**
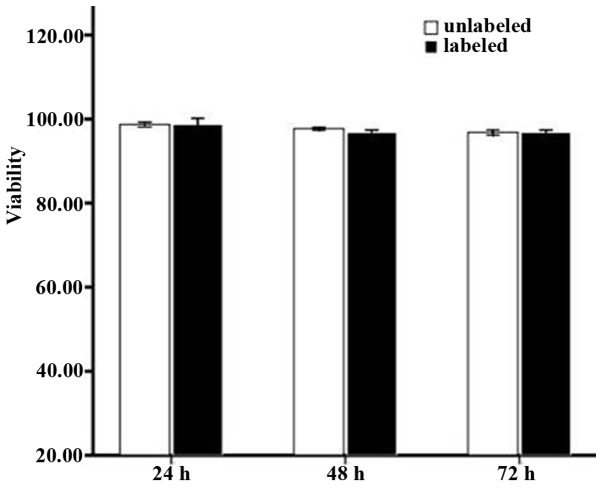
Determination of cell viability. No significant difference was observed between the labeled BMSCs and the unlabeled BMSCs after 24, 48 or 72 h. BMSC, bone marrow stromal cell.

Evaluation of cell viability by trypan blue exclusion assessment revealed an initial transient reduction in the number of cells. No significant difference was observed in the cell viability between the unlabeled and labeled cells (P>0.05) 24, 48 and 72 h after labeling (Fig. 4). In the MTT-based proliferation assay, no statistically significant decrease was observed in the proliferation of the labeled cells compared with that of the unlabeled MSCs (P>0.05) 12, 24 and 48 h after labeling (Table I).

### In vitro and in vivo MRI

**Figure 5:**
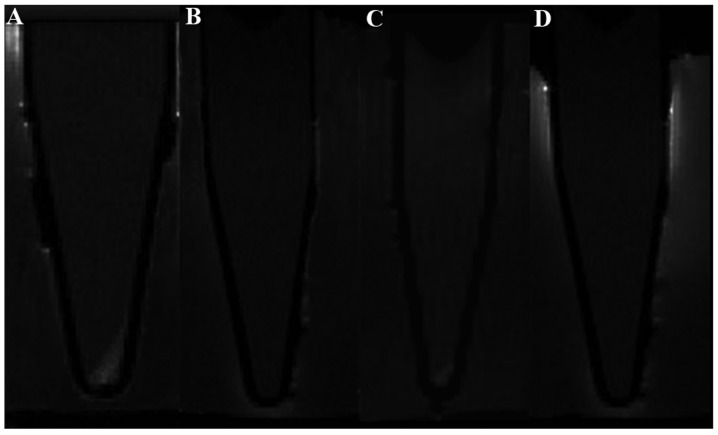
Imaging of cells using magentic resonance *in vitro*. The T1-weighted spin echo image revealed that the signal intensity of (A) the group containing Gd-DTPA, effectene and bone marrow stromal cells was markedly higher compared with that of (B) the control groups or groups treated with (C) Gd-DTPA or (D) effectene only. Gd-DTPA, gadolinium diethylenetriamine pentaacetic acid.

**Figure 6:**
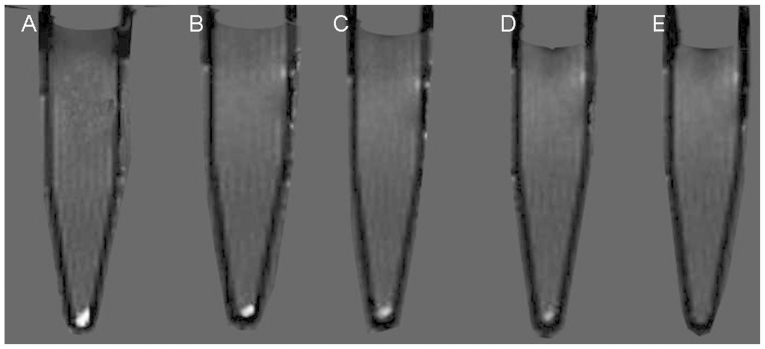
Tag persistence of BMSCs following labeling. As cell proliferation occurred, the T1-weighted image signal intensity of the BMSCs cells gradually decreased. (A-D) First, second, third and fourth cellular generation and (E) unlabeled cells, respectively. BMSC, bone marrow stromal cell.

As shown in Fig. 5, the labeled cells (Fig. 5A) demonstrated markedly higher signal intensity in T1-weighted imaging compared with the unlabeled cells (Fig. 5B) and the cells incubated with either Gd-DTPA (Fig. 5C) or effectene alone (Fig. 5D). This signal feature was consistent with the presence of Gd-DTPA particles inside the cytoplasm. Subsequently, the increased signal intensity of T1-weighted imaging of the labeled cells was evaluated and the T1 values of the labeled cells were further measured. In T1-weighted imaging, the signal intensities of the labeled cells, the cells with Gd-DTPA, the cells with effectene and the unlabeled cells were 1067±34, 635±16, 603±19 and 598±25, respectively. The signal intensity of the labeled cells was increased significantly compared with that of the negative control cells (P<0.001) and the signal intensity of the labeled cells was 1.48 times higher than that of the unlabeled cells. The T1 values of the labeled cells, the cells with Gd-DTPA, the cells with effectene and the unlabeled cells were 886±269, 2,086±58, 2,182±280 and 2,185±200 ms, respectively. The T1 value of the labeled cells was decreased significantly compared with those of the negative controls and the unlabeled cells (P<0.001). The T1 values of the labeled cells were 2.46 times lower than those of the unlabeled cells. In terms of cell proliferation, the T1-weighted image signal intensity of the BMSCs reduced gradually and, at the fifth generation, the signal intensity was similar to that observed in the unlabeled cells (Fig. 6).

**Figure 7:**
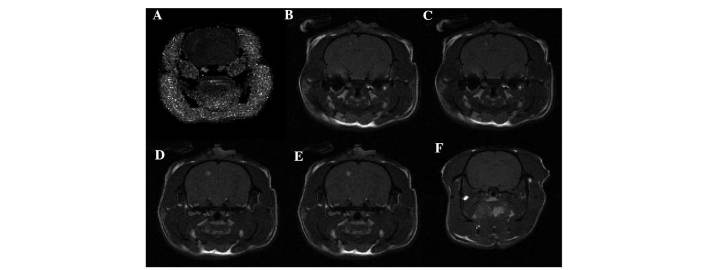
(A) Diffusion-weighted imaging map following cerebral infarction. (B-E) T1-weighted image map following stem cell injections after 1, 3, 5 and 7 days; (F) T1-weighted image map of rats following cerebral infarction and injection of unlabeled bone marrow stromal cells.

Examination of the migration of labeled cells in the *in vivo* model revealed points of high signal, which became more marked with increasing time. The scope, which was a high signal area in basal ganglia in T1-weighted, also increased gradually with time in the MRI (Fig. 7). The unlabeled control BMSCs lacked these points of increased signal intensity.

## Discussion

The development of tissue replacement by stem cell transplant or transgene therapy provides a promising treatment strategy for various diseases (3). In several mouse and primate models of diseases of the central nervous system, including Parkinson’s disease, Alzheimer’s disease, spinal cord injury and ischemic stroke, the use of exogenous BMSCs in cellular replacement therapy has achieved initial successes (33–35). However, the distribution and migration of stem cells *in vivo* has been confined to tissue analysis *in vitro* and extensive clinical application is difficult (36).

MRI provides a potent and versatile tool for non-invasive investigation. By obtaining useful information in preclinical settings, this approach generates data whose interpretation provides more informative and reliable results (8). 7T MRI have a higher spatial resolution, magnetic field intensity and tissue resolution and therefore a shorter imaging time, which is of advantage compared with other imaging techniques which include some low field intensity MRI, such as 0.3T, 1.0T, 1,5T, and 3,0T.

An understanding of the migratory behavior of stem cells following transplantation into the host environment using a non-invasive, non-toxic labeling technique in living subjects is required. Previous studies have labeled different cell populations in humans, including lymphocytes, monocytes, progenitor cells and embryonic cells, using iron-containing MR CAs, including superparamagnetic iron oxide or ultrasmall paramagnetic iron oxide (37,38). However, the incorporated particles induce signal loss due to the effects of magnetic susceptibility and, at a site of primary or secondary hemorrhage, including hemorrhagic transformation of an ischemic stroke or tumor, the physiologically iron-loaded erythrocytes induce the same loss of signal as that observed in the iron-labeled stem cells. Thus, it is not possible to distinguish between labeled stem cells which have migrated and hemorrhage (39). In addition, iron may be toxic upon excessive accumulation (40,41). A T1-weighted positive stem cell-labeled imaging technique has been previously studied *in vitro* using an established gadolinium chelate, Gd-DTPA, and uptake transfection agents, including lipofectin, to promote cellular uptake compared with that of other imaging agents (36).

Gd-DTPA is a widely used MR CA, which has been approved by the U.S. Food and Drug Administration (6). Gd-DTPA is hydrophilic and is therefore not taken up spontaneously by cells. This limitation was overcome in the present study, by the incorporation of Gd-DTPA into cationic liposomes, which enabled efficient uptake into cells by binding to anionic moieties in the cell membrane. In the present study, the transfection agent effectene was used to induce the migration of Gd-DTPA into stem cells as it has a lower toxicity and is more effective for transfection into primary cells than calcium phosphate, liposome or viral vectors (42,43). No difference was observed in the cell viability or proliferation between the labeled and unlabeled MSCs. Therefore, labeling rat MSCs with Gd-DTPA particles did not alter the cell viability or proliferation. The Gd-DTPA labeling efficiency, determined using spectrometry, was high and, indicating that Gd-DTPA labeling was successfully achieved in the BMSCs. Electron microscopy also confirmed the presence of Gd-DTPA particles inside the cytoplasm. Examination of the longevity of the CA revealed that it was retained up to the fifth generation following labeling *in vitro*. However, a major disadvantage of using Gd-DTPA was its relatively high threshold, which can be detected using MRI (44). Despite this relatively high threshold, the observed high MRI signal intensity of labeled stem cells, including those treated with Gd-DPTA, renders these labeling techniques suitable to be investigated for use in clinical applications (45).

In conclusion, the present study demonstrated a readily available strategy to magnetically label MSCs. This approach did not require any novel synthesis and therefore provided a simple and straightforward method of magnetically labeling stem cells to track their extent of migration *in vivo* and *in vitro* following implantation. In addition, as the presented labeling method uses a clinical standard agent, this may facilitate the introduction of MR monitoring of the biodistribution of magnetically labeled cells in a clinical setting. Therefore, use of the presented labeling method for MRI tracing may become a powerful tool in monitoring the transformation and distribution mechanism of transplanted cells *in vivo*.

## Figures and Tables

**Table I:** Results of the cell viability MTT assay.

	12 h		24 h		48 h	
			
Group	Labeled	Unlabeled	Labeled	Unlabeled	Labeled	Unlabeled
1	0.293	0.256	0.516	0.491	0.458	0.606
2	0.370	0.408	0.591	0.546	0.286	0.246
3	0.389	0.353	0.631	0.609	0.392	0.439
4	0.362	0.388	0.440	0.516	0.374	0.347
5	0.295	0.353	0.561	0.559	0.348	0.380
6	0.381	0.349	0.622	0.588	0.293	0.433
7	0.257	0.262	0.437	0.515	0.286	0.249
